# Transvenous Lead Extraction Using Mechanical Rotational Dilator Sheaths: A 19-Year Single-Center Experience from a Pediatric Cardiology Center

**DOI:** 10.3390/jcdd13060253

**Published:** 2026-06-07

**Authors:** Hayrettin Hakan Aykan, Musa Öztürk, Yasemin Nuran Dönmez, İlker Ertuğrul, Alpay Çeliker, Tevfik Karagöz

**Affiliations:** 1 Division of Pediatric Cardiology, Department of Pediatrics, Faculty of Medicine, Hacettepe University, Ankara 06100, Türkiye; mozturk91@gmail.com (M.Ö.); ilker.ertugrul@hacettepe.edu.tr (İ.E.); tkaragoz@hacettepe.edu.tr (T.K.); 2 Life Support Center, Hacettepe University, Ankara 06100, Türkiye; 3 Department of Pediatric Cardiology, Ankara Training and Research Hospital, Ankara 06100, Türkiye; yaseminnurandonmez@gmail.com; 4 Department of Pediatric Cardiology, VKV American Hospital, İstanbul 34365, Türkiye; alpayceliker@gmail.com

**Keywords:** pediatric cardiology, transvenous lead extraction, cardiac implantable electronic devices, mechanical dilator sheath, procedural complications

## Abstract

The increasing use of cardiac implantable electronic devices (CIEDs) in pediatric and adolescent populations has led to a growing need for transvenous lead extraction (TLE). However, data on long-term outcomes remain limited. This study aimed to evaluate the efficacy and safety of TLE using mechanical rotational dilator sheaths in a pediatric cohort. This retrospective single-center study included 35 patients who underwent TLE between 2007 and 2025. Outcomes were compared between Evolution^®^ (Cook Medical, Bloomington, IN, USA) and TightRail™ (Spectranetics/Philips, Colorado Springs, CO, USA) sheath systems. A total of 40 leads were extracted (mean age at extraction: 15.1 ± 4.2 years; 57% male). The most common indication for extraction was lead fracture/dysfunction (22/35–63%). Complete success with the procedure was achieved in 23 (66%) patients, and clinical success in 30 (86%). Major complications requiring surgery occurred in 5 (14%) patients, and minor complications in 2 (6%). Notably, all major complications occurred in patients with implantable cardioverter-defibrillator (ICD) leads (*p* = 0.013), including innominate vein injury, pericardial effusion, tricuspid entrapment, and cardiac perforation. A comparison of the Evolution^®^ (*n*:20) and TightRail™ (*n*:15) sheath groups showed no statistically significant differences in complete procedural success (*p* = 0.603), clinical success (*p* = 0.604), or the incidence of major complications (*p* = 0.640). No procedure-related mortality was observed. TLE using mechanical rotational dilator sheaths in pediatric patients is feasible and provides acceptable clinical success rates. However, the risk of major complications remains considerable, particularly in patients with ICD leads. These findings highlight the importance of careful procedural planning and performing TLE in experienced centers with immediate surgical backup.

## 1. Introduction

Improved survival among children with congenital heart disease (CHD) has led to a substantial increase in the use of implantable cardioverter-defibrillators (ICDs) and pacemakers. This growing need reflects both the inherent arrhythmic burden of CHD and complications related to surgical or interventional procedures. In parallel, advances in early diagnosis and therapeutic strategies have further broadened the use of cardiac implantable electronic devices (CIEDs), particularly in pediatric patients with primary arrhythmic disorders.

Despite the clear benefits of CIEDs, a growing number of long-term complications have been recognized, including device-related infections, venous thrombosis or occlusion, embolic events, and lead malfunction or fracture. These challenges are further compounded by the children’s ongoing somatic growth and the requirement for repeated procedures throughout follow-up [1]. In this context, transvenous lead extraction (TLE) has emerged as an effective and increasingly preferred technique for managing such complications. Although significant advancements have been made in device technology and procedural techniques, TLE continues to present both technical and clinical challenges [2]. Importantly, the selection of appropriate extraction approaches and tools plays a critical role in optimizing procedural success and minimizing complications. This consideration becomes particularly essential in patients with congenital heart disease, where diverse and complex cardiac anatomies may influence procedural outcomes.

Given the relative scarcity of long-term data on TLE in pediatric and adolescent populations, especially from high-volume centers with extensive clinical experience, further investigation is essential to inform best practices. This study aims to present a comprehensive retrospective analysis of our two-decade single-center experience with TLE, emphasizing procedural indications, clinical outcomes, and associated complications in a heterogeneous cohort of children and young patients.

## 2. Materials and Methods

### 2.1. Study Population and Demographics

**Figure 1:**
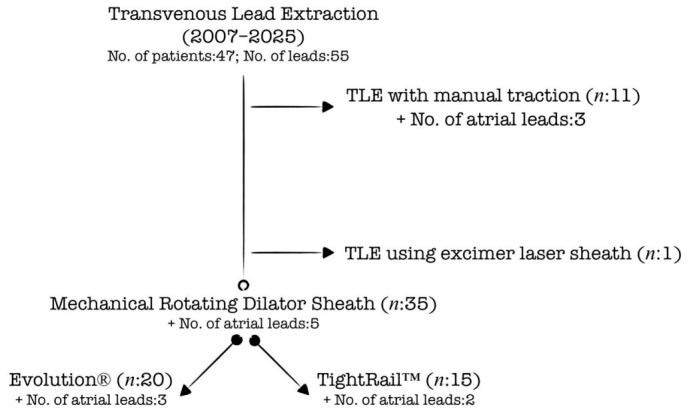
Flowchart of patient enrollment and distribution of transvenous lead extraction techniques between 2007 and 2025. The ‘*n*’ values in brackets indicate both the number of patients and ventricular leads.

This retrospective study was conducted in the pediatric cardiac catheterization laboratory at Hacettepe University Faculty of Medicine Hospital. The study included patients who underwent CIED implantation in the pediatric age group (<18 years), were clinically followed by the pediatric cardiology department, and had undergone a TLE procedure. Patients included children, adolescents, and young adults (under 25 years) at the time of extraction. Of the 47 patients who underwent transvenous lead extraction between 2007 and 2025, 35 in whom a mechanical rotational dilator sheath was used were eligible for inclusion in the analysis. The exclusion criteria are detailed in Figure 1. In total, 40 leads were targeted for extraction in 35 patients. All patients had ventricular leads, and 5 patients had an additional atrial lead. Demographic and clinical characteristics, details regarding pacemaker systems and leads, indications for TLE, procedural details, outcomes, and complications were systematically analyzed. This study was approved by the Hacettepe University Health Sciences Research Ethics Committee (research no: SBA 25/624, approval no: 2025/14-34 approval date: 8 July 2025).

### 2.2. Patient Preparation

All lead extractions were performed in the pediatric cardiac catheterization laboratory under general anesthesia, with continuous hemodynamic monitoring. A multidisciplinary team—including an electrophysiologist, an interventional cardiologist, and a cardiovascular surgeon—was present to manage potential complications, and blood products had been cross-matched in advance. In pacemaker-dependent patients, temporary pacing support was provided by advancing a 6-Fr transvenous temporary pacing electrode catheter (Boston Scientific, Marlborough, MA, USA) into the right ventricle via the femoral venous approach during lead extraction. Strict aseptic technique was employed, consisting of sequential chlorhexidine and povidone-iodine skin preparation followed by sterile draping of the generator pocket.

### 2.3. Lead Extraction Procedure

The generator pocket was opened, and the leads were disconnected from the pacemaker. A guidewire was inserted through the lead lumen to assess patency. Initially, lead removal was attempted using manual traction, counter-pressure, or counter-traction techniques. A mandrel was advanced to ensure lead integrity, and gentle traction was applied to evaluate tissue adhesion. When simple manual traction was unsuccessful, a locking stylet and hand-powered mechanical dilator sheaths (e.g., TightRail™ [Spectranetics/Philips, Colorado Springs, CO, USA] or Evolution^®^ [Cook Medical, Bloomington, IN, USA]) were employed. These devices were used to minimize the risk of lead fragmentation and to dissect fibrotic adhesions along the lead course. Device selection was primarily determined by device availability in accordance with national reimbursement policies. During extractions performed with the Cook Evolution^®^ system (Cook Medical, Bloomington, IN, USA), a dedicated telescoping sheath was routinely utilized as part of the extraction system. In contrast, in later procedural experience with the TightRail™ system (Spectranetics/Philips, Colorado Springs, CO, USA), lead extraction was performed without an additional telescoping sheath. During the procedure, a catheter was advanced through the femoral vein, and intermittent injections were administered to monitor for extravasation. In six patients with a free-floating lead remnant after extraction, the fragment was successfully retrieved from the inferior vena cava via a femoral venous approach using snare systems, including a One Snare^®^ (Merit Medical, South Jordan, UT, USA) and a Needle’s Eye Snare^®^ (Cook Medical, Bloomington, IN, USA). In one of these six patients, a jugular approach via the superior vena cava was required due to an unsuccessful attempt through the inferior vena cava. In this case, the snaring procedure was performed using a 5.2-Fr bioptome (Cook Medical, Bloomington, IN, USA), which was advanced through the jugular venous access to grasp and retrieve the lead fragment.

Particularly in procedures performed in recent years, a guidewire was advanced to the SVC via femoral venous access prior to the procedure. A 24-mm compliant sizing balloon catheter was prepared before sheath placement as a precaution against potential emergency complications, and the procedure was then initiated. Additionally, in suitable patients, transesophageal echocardiography was used to assess tricuspid valve status and the presence of intracardiac fibrous tissue or thrombus.

### 2.4. Procedural Outcomes

Complete procedural success was defined as the removal of all targeted leads and lead material from the circulatory system without causing permanent impairment or procedure-related mortality. Clinical success was defined as the complete removal of all targeted leads and lead material from the vascular system, or the retention of a small segment of the lead (<4 cm) that does not negatively impact the procedure’s goals. This may include the tip of the lead or a small segment, provided that the remaining part does not increase the risk of perforation, embolic events, ongoing infection, or cause any adverse effects. “Failure” has been defined as the inability to achieve either complete procedural or clinical success, or the occurrence of any permanent serious complication or death associated with the procedure. The procedure’s success rate was evaluated based on the number of patients.

Major complications were defined as life-threatening events requiring immediate intervention, such as respiratory or cardiac arrest, pericardial effusion, vascular laceration, hemothorax, valvular injury, or embolism. Minor complications were incidents that did not compromise the patient’s hemodynamic stability but still required medical management. Patients who developed major complications (including those requiring surgery) were considered to have undergone a ‘TLE’ procedure failure.

In cases of lead malfunction or failure, a new lead system was implanted through the same venous access. However, if a device-related infection was present, the contralateral subclavian vein was preferred. For infections requiring extraction, the generator pocket was extensively debrided, irrigated with antibiotic solutions, and a new device was implanted on the opposite side. Before discharge, all patients underwent a clinical evaluation, including transthoracic echocardiography, chest radiography, and electrocardiography on the first postoperative day to check for procedure-related complications.

### 2.5. Statistical Analysis

Statistical analyses were conducted using SPSS version 26.0 (IBM Corp., Armonk, NY, USA). Normality of continuous variables was assessed with the Shapiro–Wilk test. Continuous data are reported as mean ± standard deviation (SD) if normally distributed and as median [interquartile range (IQR), 25th–75th percentiles] if not. Categorical variables are presented as frequencies and percentages. To compare continuous variables between two independent groups, the Independent Samples *t*-test was applied for normally distributed data. For data that did not meet normality assumptions, the Mann–Whitney U test was used. Categorical variables were analyzed with the Pearson Chi-square test, with Fisher’s Exact test preferred when any cell’s expected frequency was below 5. A *p*-value of less than 0.05 was considered statistically significant.

Given the low number of major complications (*n* = 5), multivariable regression was not performed to avoid overfitting and to preserve the reliability of the statistical model. The effects of potential risk factors on clinical outcomes were evaluated using univariable analyses.

## 3. Results

A total of 35 patients were included in the study cohort. The study population consisted of 20 males (57%), with a mean age of 15.1 ± 4.2 years and a mean weight of 53.3 ± 17.8 kg. Detailed demographic and clinical characteristics of the cohort are presented in Table 1. The majority of patients had intrinsic conduction system disease, cardiomyopathy, or inherited arrhythmia/channelopathy syndromes in the setting of structurally native hearts (*n* = 27, 77%), whereas 8 patients (23%) had a history of congenital heart surgery.

The primary condition was intrinsic conduction system diseases with native structure or cardiomyopathy in the majority of patients (*n* = 27, 77.1%), while 8 patients (23%) had a history of cardiac surgery. The underlying diagnoses, listed in descending order of frequency, included surgically repaired tetralogy of Fallot (*n* = 3), Senning-repaired transposition of the great arteries (*n* = 1), congenitally corrected transposition of the great arteries with a surgically repaired ventricular septal defect (*n* = 1), common atrium with concomitant bicuspid aortic valve requiring aortic valve replacement (*n* = 1), ventricular septal defect (*n* = 1), and atrioventricular septal defect (*n* = 1). Two additional patients, who had undergone transcatheter closure for atrial septal defect and patent ductus arteriosus, presented with concomitant congenital complete atrioventricular block.

Regarding CIEDs, 19 patients (54.3%) possessed pacemaker, and 16 patients (45.7%) had ICDs. The mean age at first device implantation was 7.6 ± 3.8 years. The primary indications for pacemakers included congenital complete atrioventricular block (CCAVB; n = 12, 34.2%), followed by postoperative AV block (PAVB; *n* = 5, 14.2%). Indications for ICDs included long QT syndrome (LQTS; *n* = 8, 22.8%), ventricular tachycardia (VT; *n* = 5, 14.2%), and catecholaminergic polymorphic ventricular tachycardia (CPVT; *n* = 3, 8.5%). The mean lead dwell time prior to extraction was 7.67 ± 4.36 years. The predominant indication for TLE was lead fracture or dysfunction, accounting for 62.8% (*n* = 22) of procedures (Table 1).

A total of 40 leads were targeted for extraction. All patients had ventricular leads, and 5 patients also had an additional atrial lead (the lead characteristics are shared in Supplementary Table S1). The pectoral approach was predominantly left-sided (n = 30, 85.7%). Mechanical rotational sheaths were used for lead extraction, with the Evolution^®^ sheath in 20 patients and the TightRail™ sheath in 15. Baseline demographic and clinical characteristics were well balanced between the groups, with no statistically significant differences in age (*p* = 0.944), weight (*p* = 0.873), sex (*p* = 0.190), or lead-dwelling time (*p* = 0.475) (Table 2). Femoral approach was required in 6 cases (17.1%) across the entire cohort, with no significant difference between the sheath groups (*p* = 0.367).

**Figure 2:**
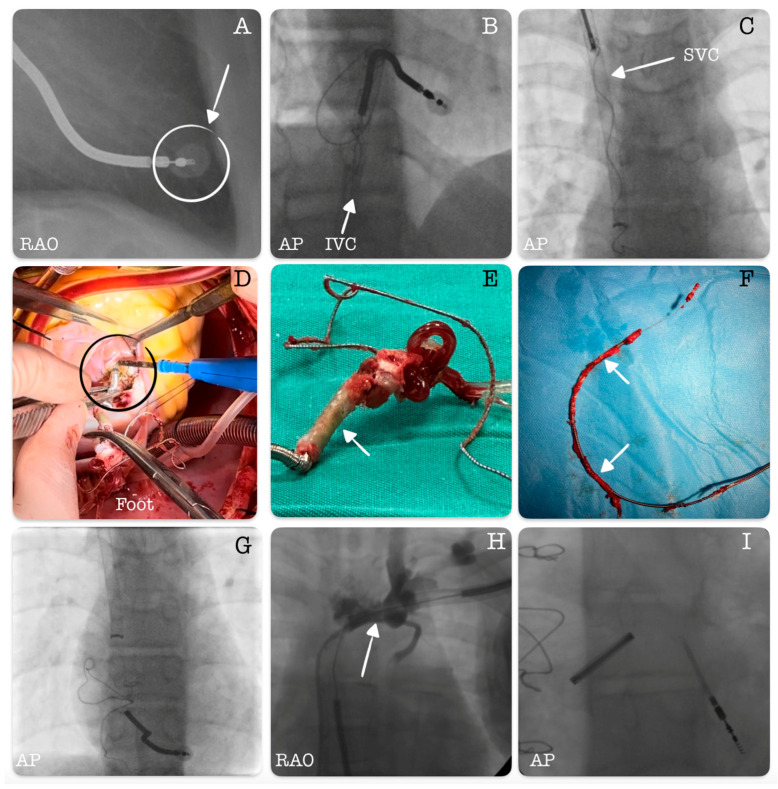
Representative fluoroscopic and intraoperative images illustrating fibrotic encapsulations, retrieval techniques, and procedural complications during lead extraction. (Fluoroscopy images are shown in anteroposterior (AP) and right anterior-oblique (RAO) views) (**A**) Fluoroscopic view demonstrating “light-bulb” shaped dense fibrotic tissue encapsulating the lead tip (white circle/arrow); (**B**) Transfemoral extraction approach using a snare/catheter advanced through the inferior vena cava; (**C**) Retrieval of migrated lead fragments using a bioptome via the superior vena cava approach; (**D**) Intraoperative visualization of the surgical removal of an ICD lead following a failed transvenous extraction attempt; (**E**,**F**) Macroscopic appearance of a calcified, dense fibrotic sheath adhering to the lead body after surgical (**E**) and transvenous (**F**) removal; (**G**) Fluoroscopic image showing fragmented and scattered lead components within the tricuspid valve and right atrium following a failed extraction attempt; (**H**) Venography demonstrating innominate vein rupture and contrast extravasation (arrow) following extraction attempt by mechanical rotational sheath; (**I**) Fluoroscopic image of a retained lead fragment in the right ventricle, which fractured during the extraction procedure in a patient who had previously undergone aortic valve replacement.

Complete procedural success was achieved in 23 patients (65.7% overall rate). Partial lead removal was feasible in 7 patients, whereas transvenous extraction failed in 5 cases (clinical success rate: 85.7%) (Table 2). Complications were observed in 7 patients (20%), of which 5 (14.3%) were major (innominate vein injury, pericardial effusion, tricuspid entrapment and cardiac perforation) and required immediate surgical intervention (Figure 2). Two patients experienced minor complications (transient bradycardia, hypotension, and minimal bleeding). Notably, all atrial leads (*n* = 5) were successfully extracted. When comparing the mechanical sheath groups, there were no statistically significant differences between the cohorts regarding complete success (*p* = 0.603), clinical success (*p* = 0.604), or the incidence of major complications (*p* = 0.640).

Univariate analysis was performed to evaluate potential predictors of major procedural complications (Table 3). The type of CIED was significantly associated with the occurrence of major complications (*p* = 0.013), with all 5 major complications occurring in patients with ICDs. Patients who developed major complications tended to be younger and have lower body weight than those without complications; however, these differences did not reach statistical significance (*p* = 0.268 and 0.383). Similarly, other clinical and procedural variables—including the presence of an atrial lead, the existence of an inferior vena cava (IVC) loop, age at first implantation, and lead dwelling time—were not significantly associated with the occurrence of major complications. Furthermore, a bulbous, mass-like fluoroscopic appearance was observed around the distal portion of the lead, likely representing fibrotic encapsulation during traction. This ‘lightbulb-like’ fibrotic structure was noted in 2 of 5 patients (Figure 2).

A subgroup analysis was conducted among patients with implantable cardioverter-defibrillator (ICD) leads (Table 4) to further explore potential risk factors within this higher-risk population. Within this subgroup, major complications were more frequently observed in patients with lower body weight and longer lead dwelling times; however, these trends did not achieve statistical significance. Notably, although not statistically significant, 4 out of 5 patients who experienced major complications had ICD leads with an SVC coil, suggesting a potentially clinically relevant association.

## 4. Discussion

The growing accessibility of pediatric-sized transvenous devices with reduced-diameter leads and smaller generators has resulted in a rising incidence of transvenous device implantation in younger pediatric patients. The increasing use of CIEDs in pediatric patients, coupled with their longer life expectancy, is leading to a higher rate of lead and device-related complications [3]. Children and adolescents often need repeated procedures for lead failure, revision, venous preservation, or infection. Although TLE is increasingly performed at specialized centers, data on its application in pediatric patients remain limited. Our study fills this gap by providing long-term follow-up on pediatric CIED cases and, importantly, comparing procedural outcomes of two mechanical rotational dilator sheaths—Evolution^®^ and TightRail™—in this high-risk population.

Pediatric TLE diverges from adult extraction in numerous significant aspects. Compared with adults, transvenous lead extraction in children is more complex, time-consuming, and technically challenging, with significantly lower rates of complete radiographic and procedural success, attributable to the formation of strong fibrous tissue surrounding the leads [4]. Recent adult TLE studies have further demonstrated the increasing procedural safety and feasibility of streamlined postprocedural management strategies in selected low-risk patients [5]. The unique physiological milieu of the pediatric cardiovascular system, characterized by smaller vessel diameters and greater elasticity, further compounds these challenges, demanding precise procedural execution and specialized instrumentation.

Data on pediatric and congenital heart disease indicate that TLE can be performed successfully at experienced centers with interdisciplinary collaboration and adequate surgical support. Cecchin et al. reported a successful extraction rate of 80% for all leads and 94% for those requiring complex extraction, using polypropylene and radiofrequency-powered sheaths, with four major and four minor complications (total 5.5%) and no mortality in a total of 144 patients and 203 leads [2]. Pham et al. recently reported a success rate of 97% for patient extractions using a laser and mechanical sheath and 98% for leads at a specialized pediatric and congenital heart disease hospital, with serious complications in 4.4% of patients and no deaths among 113 patients who underwent TLE with 162 leads [6]. Heck et al. demonstrated that TLE using hand-powered bidirectional rotating dissection sheaths in pediatric patients achieved total procedural success in 64% and clinical success in 96% of cases, with no significant complications or procedure-related mortality in extractions of 31 leads in 22 patients [7].

Our cohort supports existing research that pediatric TLE is feasible; however, it remains a challenging technique with considerable risks. In our series, complete success was achieved in 65% of patients, clinical success in 86%, and extraction failure occurred in 14%. Procedure-related complications were documented in 20% of patients, including major complications necessitating surgical intervention in 14%. The findings endorse the notion that, although satisfactory clinical success can be achieved in children and adolescents, outcomes might be lower than those reported in other studies, potentially due to a higher proportion of high-risk features in our cohort, including the presence of ICD leads, frequent use of SVC coils, and the inherent anatomical and size-related challenges of smaller pediatric patients, in addition to lead characteristics and dwell time. Advanced lead age is a recognized factor influencing extraction difficulty, while ventricular lead positioning, the presence of multiple leads, and a younger implantation age have also been linked to increased procedural complexity [2]. In a pediatric congenital heart disease cohort, Pham et al. demonstrated that the combination of implantation at age ≤ 12 years and lead age ≥ 7 years was associated with an increased likelihood of complex lead extraction, reflecting greater procedural difficulty [6].

While lead dwell time is typically considered a key component influencing extraction complexity, our data indicate that, in pediatric TLE, lead-related and anatomical factors may surpass the significance of chronological lead age [6]. One of the most clinically relevant findings of our study is the strong association between ICD leads and major procedural complications. All major complications occurred in patients with ICD leads, supporting the concept that high-voltage leads pose a greater risk for transvenous lead extraction in children. This observation is biologically plausible, as high-voltage leads, especially those with shock coils, tend to form more extensive fibrotic adhesions and are typically considered more difficult to release than conventional pacing leads [8]. The formation of ‘lightbulb’-like fibrotic tissue at the lead tip, observed in two of our patients, suggests that these patients developed an intense fibrotic reaction to the lead. While this is an unusual observation, it should be kept in mind that the probability of failure is high in this patient group (Figure 2).

Furthermore, although not statistically significant, patients in our cohort who experienced complications tended to be older at first implantation and have shorter dwell times. This seemingly paradoxical situation can be clarified by indication bias and the distinct physical properties of ICD leads. In pediatric patients, ICDs are generally implanted in late childhood or adolescence for primary or secondary prevention of sudden cardiac death, whereas conventional pacing leads are frequently implanted at younger ages. In this context, dwell time alone may not adequately reflect procedural risk in pediatric populations. In parallel, another notable trend was the high prevalence of SVC coils among patients with major complications (4 out of 5 cases). The presence of an SVC coil may further increase extraction complexity due to its larger surface area, greater endothelial contact, and propensity for dense fibrotic adhesion within the SVC—an anatomically vulnerable region during lead extraction. This risk may be further amplified in pediatric patients, where smaller vessel diameters and increased vascular elasticity can intensify the mechanical stress during sheath advancement and lead dissection. In addition, within the ICD subgroup, patients who experienced complications tended to have lower body weight and longer lead dwelling times, although these factors did not achieve statistical significance. These findings suggest that procedural risk in pediatric TLE is likely multifactorial and not solely dependent on lead age. Instead, the interplay between lead characteristics (such as ICD design and coil configuration), patient size, and anatomical constraints appears to be critical.

Taken together, these observations highlight the need for heightened procedural caution in pediatric patients with ICD leads—particularly those with SVC coils. While our sample size limits definitive conclusions, these signals may have important implications for risk stratification and procedural planning, and warrant further validation in larger, multicenter studies. From a longer-term lead management perspective, emerging low-profile lumenless lead technologies—such as the 4.1-Fr lumenless pacing lead (e.g., Medtronic SelectSecure 3830 lead) and novel small-diameter defibrillation leads (e.g., Medtronic OmniaSecure ICD lead)—may help mitigate lifetime transvenous lead burden in selected patients. For lumenless pacing leads, recent extraction series have demonstrated high procedural success with low complication rates, while pediatric experience suggests more favorable venous and valvular profiles compared with conventional leads [9,10]. However, although early clinical data on lumenless defibrillation leads are encouraging, direct evidence that these systems reduce future TLE requirements or extraction-related complications—particularly in pediatric populations—remains limited [11].

Another key aspect of our experience is the use of hand-powered mechanical dilator sheaths, including Evolution^®^ and TightRail™, along with adjunctive femoral/jugular snaring techniques in selected cases (Figure 2). Data regarding pediatric use of rotating mechanical systems remain limited; however, prior studies, such as the study by Heck et al. have demonstrated that these devices can be safely and effectively used in specialized centers [7]. Our experience supports the evidence that mechanical sheath-based extraction is technically feasible in a diverse pediatric population, including patients with congenital heart disease, cardiomyopathy, conduction system disease, and inherited arrhythmia/channelopathy syndromes. The absence of statistically significant differences in complications and procedural success between the Evolution^®^ and TightRail™ groups can be attributed to the limited sample size. The statistical instability observed in our logistic regression model, marked by high odds ratios and undefined confidence intervals, suggests limited data and complete separation rather than a genuine lack of clinical associations. Furthermore, some comprehensive adult studies have shown similar efficacy and safety in both groups [12].

The fact that all major complications in our series (11%) necessitated emergent surgical intervention underscores the inherent procedural risk of pediatric TLE, particularly in higher-risk subsets. While traditional risk factors such as dwell time are often emphasized, our findings suggest that lead-specific characteristics may be more important in pediatric patients. Notably, all major complications occurred in patients with ICD leads, most with SVC coils, indicating that lead design—together with smaller vessel size—may increase the risk of vascular or cardiac injury during extraction. Therefore, procedural risk assessment in pediatric TLE should extend beyond conventional parameters and incorporate device-related characteristics, especially in patients with ICD leads and SVC coils. From a clinical perspective, such patients may represent a higher-risk subgroup and benefit from meticulous preprocedural planning, a multidisciplinary approach, and the availability of immediate surgical backup. Accordingly, pediatric TLE should be performed in experienced centers with established extraction expertise, full anesthetic and hemodynamic support, and ready access to cardiothoracic surgery, in line with current consensus recommendations and best practices.

## 5. Conclusions

Our research contributes to the limited literature on pediatric TLE by showing that extraction using mechanical dilator sheaths is feasible and can achieve acceptable clinical success rates when performed in experienced centers. However, the non-negligible risk of major complications—particularly in patients with ICD leads—highlights the need for meticulous procedural planning and multidisciplinary management. Future multicenter studies are warranted to better define risk stratification and optimize procedural strategies in this vulnerable population.

## Figures and Tables

**Table 1:** Demographic and Clinical Characteristics of the Study Population.

Variables	Patients (*n*:35)
Sex, male, *n* (%)	20 (57%)
Age at lead extraction (years) ^1^	15.1 ± 4.2 (7.1–25.1)
Weight at lead extraction (kg) ^1^	53.3 ± 17.8 (5–82)
First implantation age (years) ^1^	7.6 ± 3.8 (1.4–17.5)
Primary Heart Condition, *n* (%)	
-Native Structure ^2^	27 (77%)
-Post-cardiac surgery	8 (23%)
Indications for pacemaker, *n* (%)	19 (54%)
-Complete congenital atrioventricular block	12 (34%)
-Postoperative atrioventricular block	5 (14%)
-Sick sinus syndrome	2 (6%)
Indications for implantable cardioverter defibrillator, *n* (%)	16 (46%)
-Long QT syndrome,	8 (23%)
-Ventricular tachycardia ^3^	5 (14%)
-Catecholaminergic polymorphic ventricular tachycardia	3 (8.5%)
Lead dwelling time (years) ^1^	7.67 ± 4.36 (1.9–16.4)
Indications for lead extraction, *n* (%)	
-Lead Fracture/Dysfunction	22 (63%)
-Infection	7 (20%)
-Dislocation/Malposition	5 (14%)
-Upgrade	1 (3%)

^1^ Data are presented as mean ± standard deviation (minimum-maximum); ^2^ Intrinsic conduction system disease, cardiomyopathy, and inherited arrhythmia/channelopathy syndromes; ^3^ Patients with ventricular tachycardia: hypertrophic cardiomyopathy (2), arrhythmogenic cardiomyopathy (1), post-surgery tetralogy of Fallot (1), idiopathic (1).

**Table 2:** Comparison of Patient Characteristics and Procedural Outcomes Between Evolution^®^ and TightRail™ Mechanical Rotational Sheaths.

Variables	Evolution^®^ (*n*:20)	TightRail™ (*n*:15)	Total (*n*:35)	*p*
Sex, male, n (%)	15 (43%)	5 (14%)	20 (57%)	0.190
Age (years) ^1^	15.1 ± 4.9	15.2 ± 3.2	15.1 ± 4.2	0.944
Weight (kg) ^1^	53.8 ± 17.7	52.8 ± 18.5	53.3 ± 17.8	0.873
Primary Heart Condition				
-Native structure ^2^	14 (40%)	13 (37%)	27 (77%)	0.419
-Post-cardiac surgery	6 (17%)	2 (6%)	8 (23%)	
Atrial lead presence	3 (8.5%)	2 (6%)	5 (14%)	0.640
Cardiac implantable electronic devices				
-Pacemaker	12 (34%)	7 (20%)	19 (54%)	0.506
-Implantable cardioverter defibrillator	8 (23%)	8 (23%)	16 (46%)	
Inferior vena cava loop	3 (8.5%)	2 (6%)	5 (14%)	0.640
First implantation age (years) ^1^	7.95 ± 3.28	7.2 ± 4.64	7.6 ± 3.8	0.475
Lead dwelling time (years) ^1^	7.2 ± 4.2	8.3 ± 4.6	7.6 ± 4.3	0.475
Pectoral approach side				
-Left	16 (46%)	14 (40%)	30 (86)	0.272
-Right	4 (11.4%)	1 (3%)	5 (14%)	
Snare system usage	2 (6%)	4 (11.4%)	6 (17%)	0.367
Procedural outcomes				
-Complete Success	13 (37%)	10 (28.6%)	23 (66%)	0.603
-Clinical Success ^3^	17 (48.6%)	13 (37%)	30 (86%)	0.604
-Failure	3 (8.6%)	2 (6%)	5 (14%)	0.640
Overall complications	3 (8.6%)	4 (11.4%)	7 (20%)	0.430
-Major complication	3 (8.6%)	2 (6%)	5 (14%)	0.640

^1^ Continuous variables were normally distributed and are presented as mean ± standard deviation. ^2^ Intrinsic conduction system disease, cardiomyopathy, and inherited arrhythmia/channelopathy syndromes. ^3^ Complete + partial success.

**Table 3:** Analysis of Clinical and Procedural Predictors Associated with Major Complications.

Variables	Clinical Success (*n*:30)	Major Complication (Requiring Surgery) (*n*:5)	*p*
Sex, male, n (%)	16 (80%)	4 (20%)	0.365
Age (years) ^1^	15.2 (9.8–24.9)	14.4 (7.1–16.1)	0.268
Weight (kg) ^1^	56 (15–79)	39 (28–73)	0.383
Primary Heart Condition			
-Native structure ^2^	22 (81.5%)	5 (18.5%)	0.315
-Post-cardiac surgery	8 (100%)	0	
Cardiac implantable electronic devices			
-Pacemaker	19 (100%)	0	0.013
-Implantable cardioverter defibrillator	11 (69%)	5 (31%)	
Presence of atrial lead	4 (80%)	1 (20%)	0.561
IVC loop presence	3 (60%)	2 (40%)	0.139
First implantation age (years) ^1^	7.7 (1.4–17.5)	9 (3.4–9.3)	0.795
Lead dwelling time (years) ^1^	6.5 (1.9–16.4)	5.5 (2.1–9.8)	0.289

IVC, inferior vena cava. ^1^ Continuous variables are presented as the median (minimum-maximum) due to their non-normal distribution. ^2^ Intrinsic conduction system disease, cardiomyopathy, and inherited arrhythmia/channelopathy syndromes.

**Table 4:** Subgroup analysis of risk factors for major complications specifically in patients undergoing ICD lead extraction.

Variables	Clinical Success (*n*:11)	Major Complication (Requiring Surgery) (*n*:5)	*p*
Sex, male, n (%)	4 (36.4%)	4 (80%)	0.282
Age (years) ^1^	14.1 (9.8–24.9)	14.4 (7.1–16.1)	0.827
Weight (kg) ^1^	56 (15–79)	39 (28–73)	0.609
First implantation age (years) ^1^	8.8 (1.4–17.5)	9 (3.4–9.3)	0.496
Lead dwelling time (years) ^1^	4.7 (1.9–16.4)	5.5 (2.1–9.8)	0.743
Presence of atrial lead	1 (9%)	1 (20%)	0.620
Double (SVC) Coil	7 (63.6%)	4 (80%)	1
Femoral Approach	0	2 (40%)	0.083
Sheath Brand			
-TightRail™	6 (54.5%)	2 (40%)	1
-Evolution^®^	5 (45.5%)	3 (60%)	

SVC, superior vena cava. ^1^ Continuous variables are presented as the median (minimum-maximum) due to their non-normal distribution.

## Data Availability

The data presented in this study are available on request from the corresponding author. The data are not publicly available due to institutional restrictions.

## Supplementary Materials

The following supporting information can be downloaded at: https://www.mdpi.com/article/10.3390/jcdd13060253/s1, Table S1: Evaluation of the clinical success of all leads undergoing transvenous lead extraction.

## Abbreviations

The following abbreviations are used in this manuscript:

ASD	Atrial Septal Defect
AVSD	Atrioventricular Septal Defect
CCAVB	Complete Congenital Atrioventricular Block
CHD	Congenital Heart Disease
CIED	Cardiac Implantable Electronic Device
CPVT	Catecholaminergic Polymorphic Ventricular Tachycardia
ICD	Implantable Cardioverter Defibrillator
IQR	Interquartile Range
IVC	Inferior Vena Cava
LQTS	Long QT Syndrome
PM	Pacemaker
PAVB	Postoperative Atrioventricular Block
PDA	Patent Ductus Arteriosus
SD	Standard Deviation
SSS	Sick Sinus Syndrome
SVC	Superior vena cava
TLE	Transvenous Lead Extraction
VT/VF	Ventricular Tachycardia/Ventricular Fibrillation
