# Supplementary material for: Transvenous Lead Extraction Using Mechanical Rotational Dilator Sheaths: A 19-Year Single-Center Experience from a Pediatric Cardiology Center
Source: J Cardiovasc Dev Dis. 2026 Jun 7;13(6):253. doi: 10.3390/jcdd13060253 (PMC13300455; doi:10.3390/jcdd13060253)
Supplement: Supplementary file 1 [file jcdd-13-00253-s001.zip › jcdd-4312768-supplementary.pdf]

Supp Table S1: Evaluation of the clinical success of all leads undergoing transvenous lead extraction

	Clinical Success (*n*: 35)	Major Complication (surgery-needed) (*n*:5)
Primary Heart Condition		
-Native Structure[1]	25	5
-Post-cardiac surgery	10	0
Cardiac implantable electronic devices		
-Pacemaker	22	0
- Implantable cardioverter defibrillator	13	5
İmplantation Chamber		
-Atrium	5	1*
-Ventricle	30	5
Inferior vena cava loop	3	2
Double (superior vena cava) Coil	7	4
First implantation age (years)[2]	7.7 [1.4-17.5]	9 [3.4-9.3]
Lead dwelling time (years)[2]	6.5 [1.9-16.4]	5.5 [2.1-9.8]
Complete success	28	-
Minor complication	2	-
Residual lead	6	-
Fibrotic residual tissue	1	-

[1]Intrinsic conduction system disease, cardiomyopathy, and inherited arrhythmia/channelopathy syndromes
[2]Continuous variables are presented as the median [minimum-maximum] due to their non-normal distribution. IVC, inferior vena cava. * Among the 5 patients who experienced major complications, 1 had a concurrent atrial lead. The ventricular lead encountered complications that necessitated surgical intervention, but the atrial lead remained untouched.
